# Crystal structure of 2-benzene­sulfon­amido-3-hy­droxy­propanoic acid

**DOI:** 10.1107/S2056989015020149

**Published:** 2015-10-31

**Authors:** Nabila Jabeen, Misbah Mushtaq, Muhammad Danish, Muhammad Nawaz Tahir, Muhammad Asam Raza

**Affiliations:** a Department of Chemistry, Institute of Natural Sciences, University of Gujrat, Gujrat 50700, Pakistan; b Department of Physics, University of Sargodha, Sargodha, Punjab, Pakistan

**Keywords:** crystal structure, benzene­sulfonamido, propanoic acid, sulfonyl group, O—H⋯O hydrogen bonds

## Abstract

In the title compound, C_9_H_11_NO_5_S, the O=S=O plane of the sulfonyl group is twisted at a dihedral angle of 52.54 (16)° with respect to the benzene ring. The dihedral angle between the carb­oxy­lic acid group and the benzene ring is 49.91 (16)°. In the crystal, C—H⋯O, N—H⋯O and O—H⋯O hydrogen bonds link the mol­ecules into (001) sheets.

## Related literature   

For related structures, see: Aguilar-Castro *et al.* (2004 ▸); Arshad *et al.* (2009 ▸, 2012 ▸); Zolotarev *et al.* (2014 ▸).
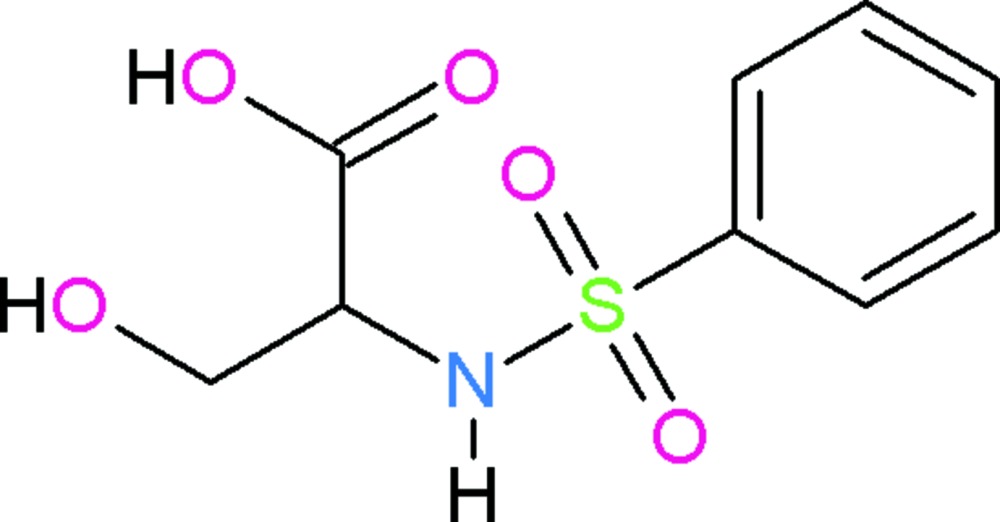


## Experimental   

### Crystal data  

C_9_H_11_NO_5_S
*M* *_r_* = 245.25
Orthorhombic, 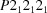

*a* = 5.0464 (4) Å
*b* = 9.9752 (8) Å
*c* = 21.4701 (17) Å
*V* = 1080.78 (15) Å^3^
*Z* = 4
Mo *K*α radiation
μ = 0.31 mm^−1^
*T* = 296 K
0.40 × 0.20 × 0.18 mm

### Data collection  

Bruker Kappa APEXII CCD diffractometer
Absorption correction: multi-scan (*SADABS*; Bruker, 2005 ▸) *T*_min_ = 0.890, *T*_max_ = 0.950
5013 measured reflections
2354 independent reflections
1978 reflections with *I* > 2σ(*I*)
*R*_int_ = 0.025

### Refinement  

*R*[*F* ^2^ > 2σ(*F* ^2^)] = 0.042
*wR*(*F* ^2^) = 0.093
*S* = 1.03
2354 reflections
149 parameters
H atoms treated by a mixture of independent and constrained refinement
Δρ_max_ = 0.21 e Å^−3^
Δρ_min_ = −0.28 e Å^−3^
Absolute structure: Flack *x* determined using 919 quotients [(*I* ^+^)−(*I* ^−^)]/[(*I* ^+^)^+^(*I* ^−^)] (Parsons *et al.*, 2013 ▸)
Absolute structure parameter: 0.05 (5)

### 

Data collection: *APEX2* (Bruker, 2007 ▸); cell refinement: *SAINT* (Bruker, 2007 ▸); data reduction: *SAINT* (Bruker, 2007 ▸); program(s) used to solve structure: *SHELXS97* (Sheldrick, 2008 ▸); program(s) used to refine structure: *SHELXL2014* (Sheldrick, 2015 ▸); molecular graphics: *ORTEP-3 for Windows* (Farrugia, 2012 ▸) and *PLATON* (Spek, 2009 ▸); software used to prepare material for publication: *WinGX* (Farrugia, 2012 ▸) and *PLATON* (Spek, 2009 ▸).

## Supplementary Material

Crystal structure: contains datablock(s) global, I. DOI: 10.1107/S2056989015020149/hb7530sup1.cif

Structure factors: contains datablock(s) I. DOI: 10.1107/S2056989015020149/hb7530Isup2.hkl

Supporting information file. DOI: 10.1107/S2056989015020149/hb7530Isup3.cml

**Fig. 1.**
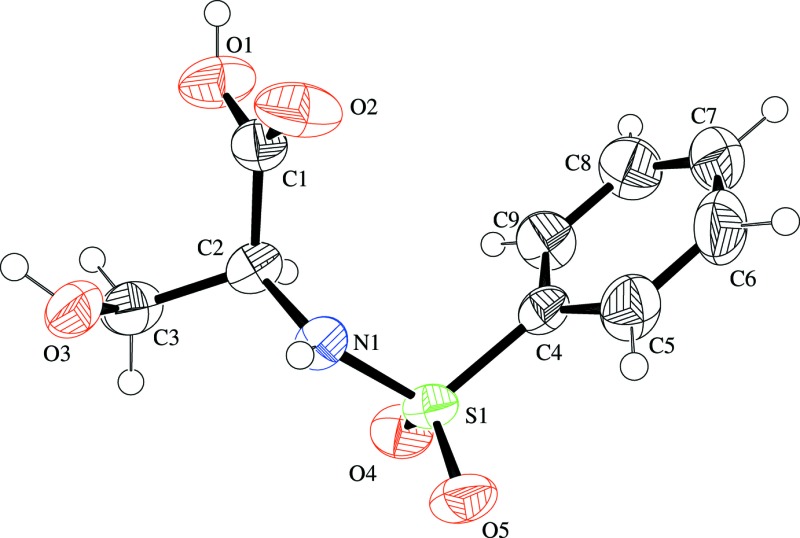
View of the asymmetric unit of title compound with displacement ellipsoids drawn at the 50% probability level.

. DOI: 10.1107/S2056989015020149/hb7530fig1.tif
View of the asymmetric unit of title compound with displacement ellipsoids drawn at the 50% probability level.

**Fig. 2.**
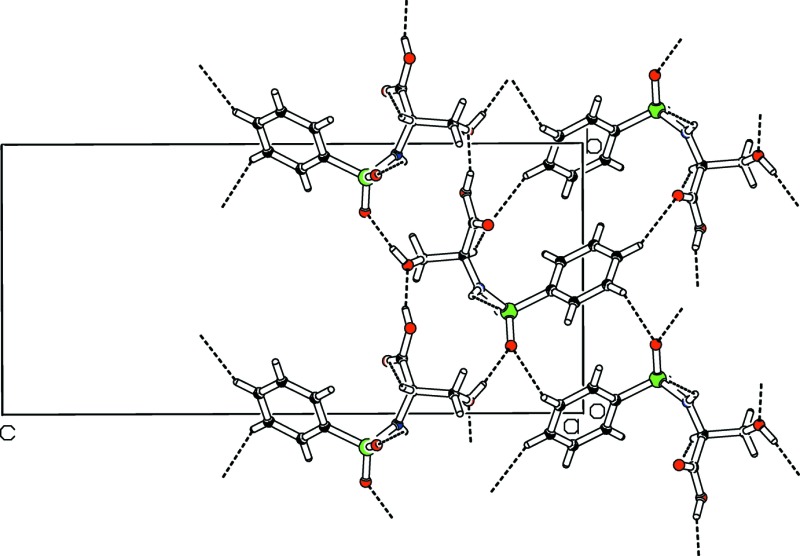
The partial packing (PLATON; Spek, 2009), which shows that molecules form two dimensional polymeric network.

PLATON . DOI: 10.1107/S2056989015020149/hb7530fig2.tif
The partial packing (*PLATON*; Spek, 2009), which shows that mol­ecules form two dimensional polymeric network.

CCDC reference: 1433189

Additional supporting information:  crystallographic information; 3D view; checkCIF report

## Figures and Tables

**Table 1:** Hydrogen-bond geometry (, )

*D*H*A*	*D*H	H*A*	*D* *A*	*D*H*A*
O1H1O3^i^	0.84(5)	1.81(5)	2.621(4)	164(5)
O3H3O5^i^	0.82	1.96	2.754(3)	164
N1H1*A*O4^ii^	0.86	2.39	3.066(4)	136
C2H2O2^iii^	0.98	2.48	3.425(5)	162
C6H6O5^iv^	0.93	2.52	3.342(5)	148
C7H7O2^v^	0.93	2.58	3.347(5)	141

Symmetry codes: (i) 
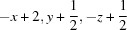
; (ii) 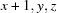; (iii) 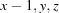; (iv) 
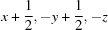
; (v) 
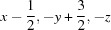
.

## supplementary crystallographic information

### S1. Comment

The title compound (I), (Fig. 1) has been synthesized for complexation and other studius.

The crystal structures of *N*-((4-methylphenyl)sulfonyl)serine (Zolotarev *et al.*, 2014), *N(S)*-(*p*-toluenesulfonyl)-*L*-alanine (Aguilar-Castro *et al.*, 2004), 2-benzenesulfonamido-3-methylbutyric acid (Arshad *et al.*, 2012) and (2*R*)-2-benzenesulfonamido-2- phenylethanoic acid (Arshad *et al.*, 2009) have been reported which are related to the title compound.

The aminoacetic acid moiety B (C1/C2/N1/O1/O2) is roughly planar with r.m.s. deviation of 0.0588 Å. The dihedral angle between the benzene ring and B is 52.96 (14)°. The sulfonyl group *C* (S1/O4/O5) is oriented at a dihedral angle of 52.54 (16)° with the parent benzene ring. In the crystal, the molecules are linked into a two-dimensional polymeric network (Table 2, Fig. 2) due to H-bondings of C–H···O, N–H···O and O–H···O types with base vectors [100], [010] and in the plane (001).

### S2. Experimental

The title compound was prepared by using equimolar ratio of *L*-serine and benzenesulfonyl chloride in 40 ml water. The benzenesulfonyl chloride disolved in distilled water was added pinch by pinch in the *L*-serine already disolved in distilled water and stirred at 296–298 K, while keeping the pH of the reaction mixture was maintained at 8–9 by adding 1.0 *M* sodium bicarbonate solution. The 1.0 *M* HCl solution was added after an hour which resulted in the form of white precipitates. The precipitates obtained were filtered and dried from which colourless needles of (I) were obtained after recrystallization from ethanol solution after 48 h. Yield: 68% Melting point: 493 K.

### S3. Refinement

The coordinates of H-atom of carboxyl group were refined. The other H-atoms were positioned geometrically (O—H = 0.82, N—H = 0.86, C–H = 0.93—0.98 Å) and refined as riding with *U*_iso_(H) = *xU*_eq_(C, N, O), where *x* = 1.5 for hydroxy and *x* = 1.2 for all other H-atoms.

### Crystal data

**Table:**

C_9_H_11_NO_5_S	*D*_x_ = 1.507 Mg m^−^^3^
*M**_r_* = 245.25	Mo *K*α radiation, λ = 0.71073 Å
Orthorhombic, *P*2_1_2_1_2_1_	Cell parameters from 1978 reflections
*a* = 5.0464 (4) Å	θ = 2.8–27.1°
*b* = 9.9752 (8) Å	µ = 0.31 mm^−^^1^
*c* = 21.4701 (17) Å	*T* = 296 K
*V* = 1080.78 (15) Å^3^	Needle, colorless
*Z* = 4	0.40 × 0.20 × 0.18 mm
*F*(000) = 512	

### Data collection

**Table:**

Bruker Kappa APEXII CCD diffractometer	2354 independent reflections
Radiation source: fine-focus sealed tube	1978 reflections with *I* > 2σ(*I*)
Graphite monochromator	*R*_int_ = 0.025
Detector resolution: 7.80 pixels mm^-1^	θ_max_ = 27.1°, θ_min_ = 2.8°
ω scans	*h* = −6→6
Absorption correction: multi-scan (*SADABS*; Bruker, 2005)	*k* = −9→12
*T*_min_ = 0.890, *T*_max_ = 0.950	*l* = −27→27
5013 measured reflections	

### Refinement

**Table:**

Refinement on *F*^2^	Secondary atom site location: difference Fourier map
Least-squares matrix: full	Hydrogen site location: inferred from neighbouring sites
*R*[*F*^2^ > 2σ(*F*^2^)] = 0.042	H atoms treated by a mixture of independent and constrained refinement
*wR*(*F*^2^) = 0.093	*w* = 1/[σ^2^(*F*_o_^2^) + (0.0432*P*)^2^ + 0.1053*P*] where *P* = (*F*_o_^2^ + 2*F*_c_^2^)/3
*S* = 1.03	(Δ/σ)_max_ < 0.001
2354 reflections	Δρ_max_ = 0.21 e Å^−^^3^
149 parameters	Δρ_min_ = −0.28 e Å^−^^3^
0 restraints	Absolute structure: Flack *x* determined using 919 quotients [(*I*^+^)-(*I*^-^)]/[(*I*^+^)^+^(*I*^-^)] (Parsons *et al.*, 2013)
Primary atom site location: structure-invariant direct methods	Absolute structure parameter: 0.05 (5)

### Special details

**Table:**

Geometry. All e.s.d.'s (except the e.s.d. in the dihedral angle between two l.s. planes) are estimated using the full covariance matrix. The cell e.s.d.'s are taken into account individually in the estimation of e.s.d.'s in distances, angles and torsion angles; correlations between e.s.d.'s in cell parameters are only used when they are defined by crystal symmetry. An approximate (isotropic) treatment of cell e.s.d.'s is used for estimating e.s.d.'s involving l.s. planes.
Refinement. Refinement of *F*^2^ against ALL reflections. The weighted *R*-factor *wR* and goodness of fit *S* are based on *F*^2^, conventional *R*-factors *R* are based on *F*, with *F* set to zero for negative *F*^2^. The threshold expression of *F*^2^ > σ(*F*^2^) is used only for calculating *R*-factors(gt) *etc*. and is not relevant to the choice of reflections for refinement. *R*-factors based on *F*^2^ are statistically about twice as large as those based on *F*, and *R*-factors based on ALL data will be even larger.

### Fractional atomic coordinates and isotropic or equivalent isotropic displacement parameters (Å^2^)

**Table:**

	*x*	*y*	*z*	*U*_iso_*/*U*_eq_	
S1	0.68841 (17)	0.37500 (8)	0.12794 (3)	0.0362 (2)	
O1	0.7864 (6)	0.8154 (3)	0.20233 (14)	0.0624 (8)	
H1	0.871 (11)	0.885 (5)	0.194 (2)	0.094*	
O2	1.1199 (6)	0.6949 (3)	0.16402 (14)	0.0651 (8)	
O3	0.9705 (7)	0.5474 (2)	0.30216 (11)	0.0583 (8)	
H3	1.0059	0.6065	0.3274	0.087*	
O4	0.4135 (5)	0.3816 (3)	0.14371 (11)	0.0504 (6)	
O5	0.8178 (6)	0.2474 (2)	0.12476 (11)	0.0502 (6)	
N1	0.8462 (6)	0.4600 (2)	0.17945 (11)	0.0368 (6)	
H1A	0.9985	0.4323	0.1920	0.044*	
C1	0.9047 (8)	0.7029 (3)	0.18768 (14)	0.0406 (8)	
C2	0.7369 (7)	0.5833 (3)	0.20543 (14)	0.0384 (8)	
H2	0.5583	0.5962	0.1886	0.046*	
C3	0.7171 (9)	0.5712 (4)	0.27649 (16)	0.0530 (10)	
H3A	0.6444	0.6533	0.2936	0.064*	
H3B	0.5990	0.4981	0.2873	0.064*	
C4	0.7318 (7)	0.4526 (3)	0.05482 (14)	0.0365 (8)	
C5	0.9275 (9)	0.4077 (4)	0.01556 (16)	0.0542 (10)	
H5	1.0338	0.3355	0.0269	0.065*	
C6	0.9645 (11)	0.4717 (4)	−0.04132 (17)	0.0653 (12)	
H6	1.0988	0.4436	−0.0679	0.078*	
C7	0.8028 (10)	0.5765 (4)	−0.05829 (16)	0.0609 (11)	
H7	0.8250	0.6180	−0.0967	0.073*	
C8	0.6103 (9)	0.6193 (4)	−0.01864 (17)	0.0618 (12)	
H8	0.5028	0.6909	−0.0301	0.074*	
C9	0.5718 (9)	0.5581 (4)	0.03840 (17)	0.0512 (9)	
H9	0.4395	0.5879	0.0652	0.061*	

### Atomic displacement parameters (Å^2^)

**Table:**

	*U*^11^	*U*^22^	*U*^33^	*U*^12^	*U*^13^	*U*^23^
S1	0.0337 (4)	0.0291 (3)	0.0459 (4)	−0.0024 (4)	−0.0051 (4)	0.0017 (3)
O1	0.061 (2)	0.0327 (13)	0.0933 (19)	0.0046 (14)	0.0105 (17)	−0.0028 (13)
O2	0.054 (2)	0.0441 (14)	0.097 (2)	0.0024 (14)	0.0283 (17)	0.0134 (14)
O3	0.089 (2)	0.0342 (13)	0.0514 (14)	0.0014 (15)	−0.0182 (15)	−0.0063 (10)
O4	0.0350 (13)	0.0529 (14)	0.0634 (14)	−0.0087 (13)	−0.0019 (13)	0.0046 (12)
O5	0.0590 (17)	0.0282 (11)	0.0635 (14)	0.0039 (11)	−0.0118 (16)	0.0015 (10)
N1	0.0325 (16)	0.0352 (14)	0.0428 (12)	0.0059 (13)	−0.0089 (14)	−0.0030 (11)
C1	0.042 (2)	0.0349 (17)	0.0444 (17)	0.0068 (17)	−0.0013 (18)	0.0026 (14)
C2	0.0300 (19)	0.0357 (16)	0.0496 (16)	0.0040 (14)	−0.0014 (16)	−0.0043 (13)
C3	0.059 (3)	0.0427 (19)	0.0569 (19)	−0.005 (2)	0.025 (2)	−0.0085 (15)
C4	0.036 (2)	0.0331 (15)	0.0405 (14)	−0.0015 (16)	−0.0064 (16)	−0.0051 (12)
C5	0.059 (3)	0.051 (2)	0.0528 (19)	0.018 (2)	0.001 (2)	−0.0045 (16)
C6	0.081 (3)	0.069 (3)	0.0457 (19)	0.011 (3)	0.014 (2)	−0.011 (2)
C7	0.080 (3)	0.062 (2)	0.0400 (16)	0.000 (3)	−0.006 (2)	0.0020 (16)
C8	0.071 (3)	0.057 (2)	0.058 (2)	0.014 (2)	−0.011 (2)	0.0106 (19)
C9	0.048 (2)	0.050 (2)	0.0560 (19)	0.0159 (19)	0.003 (2)	0.0046 (17)

### Geometric parameters (Å, º)

**Table:**

S1—O4	1.429 (3)	C3—H3A	0.9700
S1—O5	1.432 (2)	C3—H3B	0.9700
S1—N1	1.605 (3)	C4—C9	1.373 (5)
S1—C4	1.764 (3)	C4—C5	1.374 (5)
O1—C1	1.310 (4)	C5—C6	1.391 (5)
O1—H1	0.84 (5)	C5—H5	0.9300
O2—C1	1.201 (4)	C6—C7	1.375 (6)
O3—C3	1.413 (5)	C6—H6	0.9300
O3—H3	0.8200	C7—C8	1.360 (6)
N1—C2	1.458 (4)	C7—H7	0.9300
N1—H1A	0.8600	C8—C9	1.382 (5)
C1—C2	1.512 (5)	C8—H8	0.9300
C2—C3	1.534 (5)	C9—H9	0.9300
C2—H2	0.9800		
			
O4—S1—O5	119.67 (16)	C2—C3—H3A	109.7
O4—S1—N1	107.09 (15)	O3—C3—H3B	109.7
O5—S1—N1	106.03 (14)	C2—C3—H3B	109.7
O4—S1—C4	108.12 (15)	H3A—C3—H3B	108.2
O5—S1—C4	106.92 (15)	C9—C4—C5	121.0 (3)
N1—S1—C4	108.64 (14)	C9—C4—S1	119.5 (3)
C1—O1—H1	115 (4)	C5—C4—S1	119.5 (3)
C3—O3—H3	109.5	C4—C5—C6	119.1 (4)
C2—N1—S1	121.4 (2)	C4—C5—H5	120.5
C2—N1—H1A	119.3	C6—C5—H5	120.5
S1—N1—H1A	119.3	C7—C6—C5	120.1 (4)
O2—C1—O1	124.8 (4)	C7—C6—H6	119.9
O2—C1—C2	124.1 (3)	C5—C6—H6	119.9
O1—C1—C2	111.1 (3)	C8—C7—C6	119.7 (4)
N1—C2—C1	110.9 (3)	C8—C7—H7	120.1
N1—C2—C3	109.8 (3)	C6—C7—H7	120.1
C1—C2—C3	110.4 (3)	C7—C8—C9	121.1 (4)
N1—C2—H2	108.5	C7—C8—H8	119.4
C1—C2—H2	108.5	C9—C8—H8	119.4
C3—C2—H2	108.5	C4—C9—C8	118.9 (4)
O3—C3—C2	110.0 (3)	C4—C9—H9	120.6
O3—C3—H3A	109.7	C8—C9—H9	120.6
			
O4—S1—N1—C2	−37.2 (3)	N1—S1—C4—C9	−83.5 (3)
O5—S1—N1—C2	−166.0 (2)	O4—S1—C4—C5	−148.5 (3)
C4—S1—N1—C2	79.4 (3)	O5—S1—C4—C5	−18.4 (3)
S1—N1—C2—C1	−114.8 (3)	N1—S1—C4—C5	95.6 (3)
S1—N1—C2—C3	122.8 (3)	C9—C4—C5—C6	0.7 (6)
O2—C1—C2—N1	−11.0 (5)	S1—C4—C5—C6	−178.4 (3)
O1—C1—C2—N1	170.0 (3)	C4—C5—C6—C7	−1.4 (6)
O2—C1—C2—C3	111.1 (4)	C5—C6—C7—C8	1.4 (7)
O1—C1—C2—C3	−68.0 (4)	C6—C7—C8—C9	−0.8 (7)
N1—C2—C3—O3	59.3 (4)	C5—C4—C9—C8	−0.1 (6)
C1—C2—C3—O3	−63.3 (4)	S1—C4—C9—C8	179.1 (3)
O4—S1—C4—C9	32.4 (3)	C7—C8—C9—C4	0.1 (7)
O5—S1—C4—C9	162.5 (3)		

### Hydrogen-bond geometry (Å, º)

**Table:**

*D*—H···*A*	*D*—H	H···*A*	*D*···*A*	*D*—H···*A*
O1—H1···O3^i^	0.84 (5)	1.81 (5)	2.621 (4)	164 (5)
O3—H3···O5^i^	0.82	1.96	2.754 (3)	164
N1—H1*A*···O4^ii^	0.86	2.39	3.066 (4)	136
C2—H2···O2^iii^	0.98	2.48	3.425 (5)	162
C6—H6···O5^iv^	0.93	2.52	3.342 (5)	148
C7—H7···O2^v^	0.93	2.58	3.347 (5)	141

Symmetry codes: (i) −*x*+2, *y*+1/2, −*z*+1/2; (ii) *x*+1, *y*, *z*; (iii) *x*−1, *y*, *z*; (iv) *x*+1/2, −*y*+1/2, −*z*; (v) *x*−1/2, −*y*+3/2, −*z*.
