# Supplementary figures and images for: Crystal structure of 2-benzene­sulfon­amido-3-hy­droxy­propanoic acid
Source: Acta Crystallogr E Crystallogr Commun. 2015 Oct 31;71(Pt 11):o902–3. doi: 10.1107/S2056989015020149 (PMC4645086; doi:10.1107/S2056989015020149)

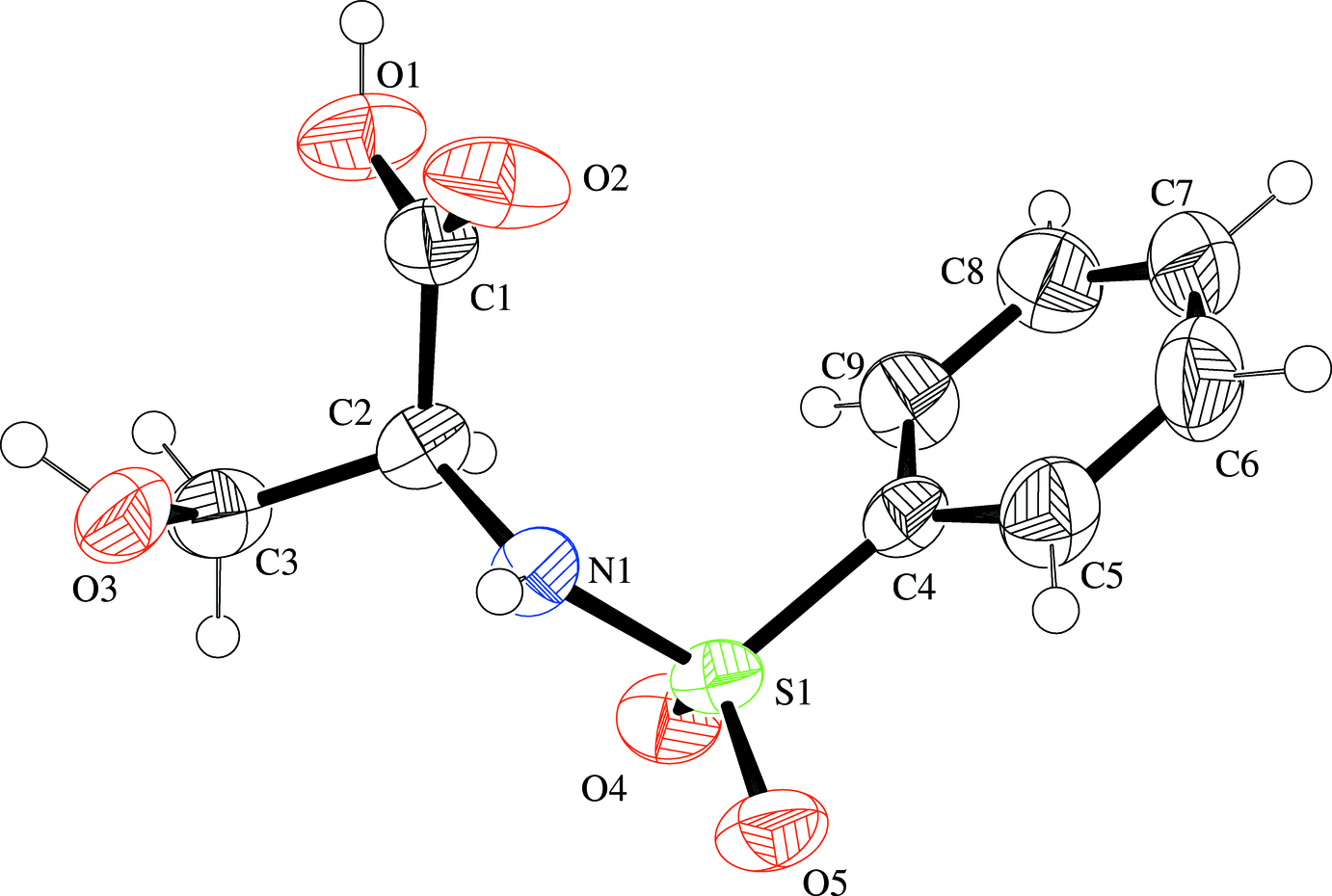

Supplement: Supplementary file 4 [file e-71-0o902-fig1.tif]

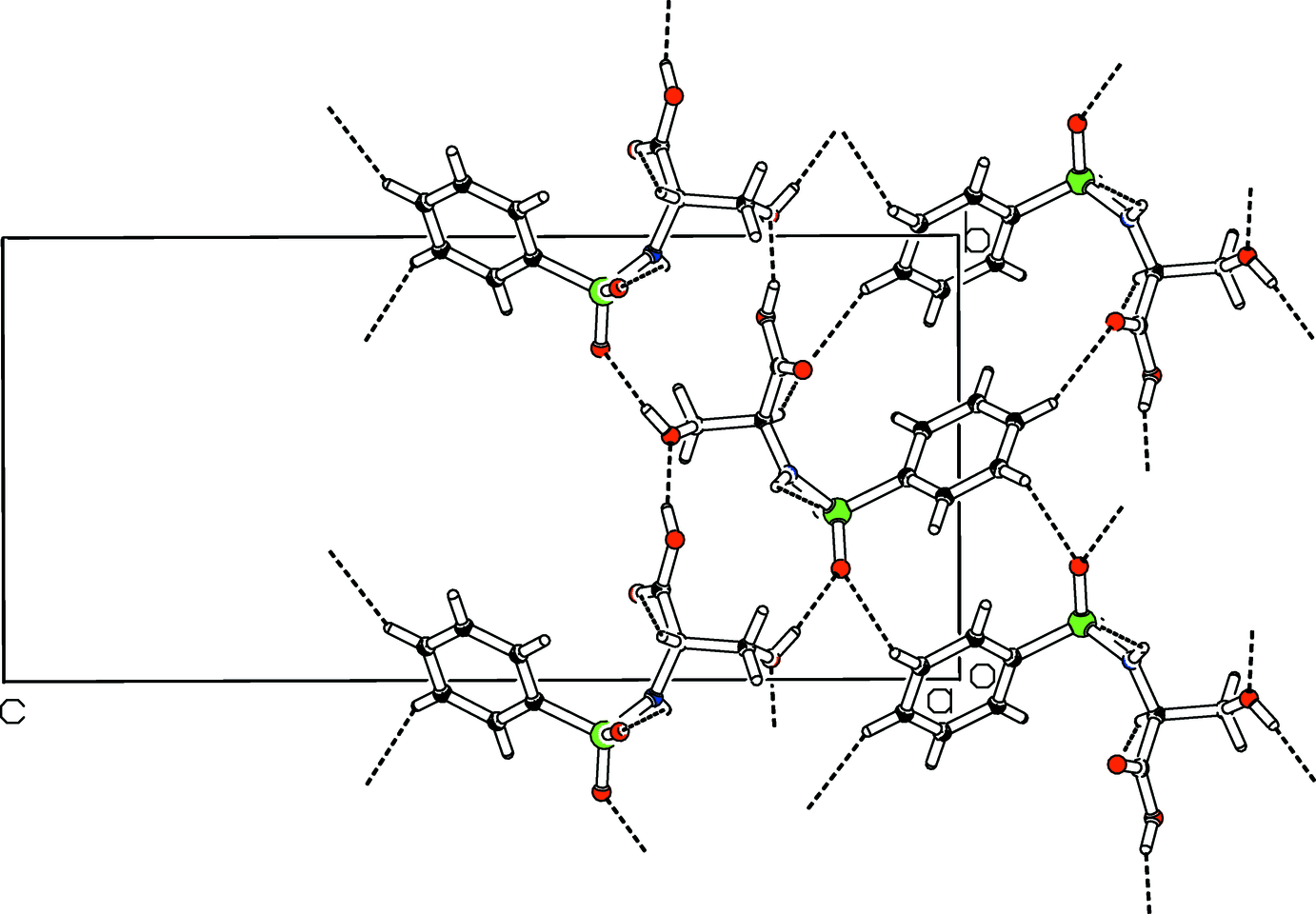

Supplement: Supplementary file 5 [file e-71-0o902-fig2.tif]
